# Best practices for the care of pregnant people living with TB

**DOI:** 10.5588/ijtld.23.0031

**Published:** 2023-05-01

**Authors:** C. Maugans, M. Loveday, S. Hlangu, C. Waitt, M. Van Schalkwyk, B. van de Water, N. Salazar-Austin, L. McKenna, J. S. Mathad, E. Kalk, R. Hurtado, J. Hughes, A. C. Eke, S. Ahmed, J. Furin

**Affiliations:** 1 Sentinel Project on Pediatric Drug Resistant Tuberculosis, Boston, MA, USA; 2 HIV and other Infectious Diseases Research Unit (HIDRU), South African Medical Research Council, Durban, South Africa; 3 Department of Pharmacology and Therapeutics, University of Liverpool, UK, and the Infectious Diseases Institute, Makerere University College of Health Sciences, Kampala, Uganda; 4 Division of Adult Infectious Diseases, Department of Medicine, Faculty of Medicine and Health Sciences, Stellenbosch University and Tygerberg Hospital, Cape Town, South Africa; 5 Boston College Connell School of Nursing, Chestnut Hill, MA, USA; 6 Department of Pediatrics, Johns Hopkins University School of Medicine, Baltimore, MD, USA; 7 Treatment Action Group, New York, NY, USA; 8 Departments of Medicine and Obstetrics & Gynecology, Center for Global Health, Weill Cornell Medicine, New York, NY, USA; 9 Centre for Infectious Disease Epidemiology & Research, School of Public Health, University of Cape Town, South Africa; 10 Division of Infectious Diseases, Massachusetts General Hospital, Harvard Medical School, Boston, MA, USA; 11 Global Health Committee, Boston, MA, USA; 12 Desmond Tutu TB Centre, Department of Paediatrics and Child Health, Faculty of Medicine and Health Sciences, Stellenbosch University, Cape Town, South Africa; 13 Division of Maternal Fetal Medicine, Department of Gynecology & Obstetrics, Johns Hopkins University School of Medicine, Baltimore, MD, USA; 14 Interactive Research and Development, Karachi, Pakistan; 15 Harvard Medical School, Department of Global Health and Social Medicine, Boston, MA, USA

**Keywords:** tuberculosis, pregnancy, support, treatment, prevention, best practice

## Abstract

**BACKGROUND:**

Each year more than 200,000 pregnant people become sick with TB, but little is known about how to optimize their diagnosis and therapy. Although there is a need for further research in this population, it is important to recognize that much can be done to improve the services they currently receive.

**METHODS:**

Following a systematic review of the literature and the input of a global team of health professionals, a series of best practices for the diagnosis, prevention and treatment of TB during pregnancy were developed.

**RESULTS:**

Best practices were developed for each of the following areas: 1) screening and diagnosis; 2) reproductive health services and family planning; 3) treatment of drug-susceptible TB; 4) treatment of rifampicin-resistant/multidrug-resistant TB; 5) compassionate infection control practices; 6) feeding considerations; 7) counseling and support; 8) treatment of TB infection/TB preventive therapy; and 9) research considerations.

**CONCLUSION:**

Effective strategies for the care of pregnant people across the TB spectrum are readily achievable and will greatly improve the lives and health of this under-served population.

## Abstract (fr)

**CONTEXTE:**

Chaque année, environ 200 000 femmes enceintes contractent la TB, mais peu de données sont disponibles pour savoir comment optimiser leur expérience diagnostique et thérapeutique. Si davantage d’études doivent être réalisées dans cette population, beaucoup peut être fait pour améliorer les services dont elle bénéficie.

**MÉTHODES:**

Une série de « bonnes pratiques » pour le diagnostic, la prévention et le traitement de la TB pendant la grossesse a été élaborée en prenant appui sur une revue systématique de la littérature, ainsi que grâce à la contribution d’une équipe internationale de professionnels de la santé travaillant sur les questions relatives à la TB chez les femmes enceintes.

**RÉSULTATS:**

Des bonnes pratiques ont été élaborées dans chacun des domaines suivants : 1) dépistage et diagnostic ; 2) services de santé génésique et planification familiale ; 3) traitement de la TB pharmacosensible ; 4) traitement de la TB résistante à la rifampicine et multirésistante ; 5) pratiques compatissantes de contrôle des infections ; 6) considérations relatives à l’alimentation ; 7) conseil et soutien ; 8) traitement de l’infection tuberculeuse/traitement préventif de la TB ; et 9) considérations relatives à la recherche.

**CONCLUSION:**

Des stratégies efficaces pour améliorer la prise en charge des femmes enceintes tout au long du spectre de la TB sont à la portée des prestataires et des programmes et sont susceptibles d’améliorer considérablement la vie et la santé de cette population mal prise en charge.

TB disease is a leading cause of global morbidity and mortality, with an estimated 10.6 million incident cases, resulting in an estimated 1.6 million deaths in 2021.^1^ More than 200,000 pregnant people become sick with TB each year and are at high risk of adverse parental, pregnancy-related and infant outcomes.^2^ Approximately 20% of the global TB disease burden occurs among people who have the potential to become pregnant, who would therefore be more vulnerable for a variety of biological and social reasons.^3^ Optimizing their care should be a global priority.^4^ However, the care of pregnant people with TB is complicated by the fact that there are limited data to guide optimal clinical management. Pregnant people have not benefitted from fair inclusion in most TB clinical trials,^5^,^6^ and as a result, clinicians are not comfortable managing them in the absence of evidence-based guidelines. This lack of experience in managing TB and its complications during pregnancy can lead to fear, discrimination and sub-optimal clinical practices.^7^ The result is that while pregnant people are more likely to become sick from TB, they are less likely to receive high-quality person-centered care, placing them, their children and their families at risk of poor outcomes.^8^

As a matter of urgency, pregnant people should be prioritized in the TB research and care agenda so that evidence-based strategies can be developed for their unique needs.^9^ This is pressing for TB programs, but also for antenatal and child health services because TB during pregnancy is associated with an increased risk of developing pre-eclampsia, eclampsia, anemia, and sepsis; and can result in intrauterine fetal growth restriction, preterm birth, small for gestational age (SGA) infants, and low birth weight.^10^,^11^ However, there is much that can be done to improve the treatment of pregnant people at every stage of the TB care cascade until data on optimal management of TB in pregnancy become available.^12^ The best way to ensure healthy parent-child pairs is through TB prevention, combined with early diagnosis of TB disease, followed by rapid initiation of effective therapy delivered through supportive and compassionate means.^13^ Here, we highlight key evidence, challenges, and best practice for TB prevention and care in pregnant people, including screening and diagnosis of TB disease; contraceptive and reproductive advice through family planning; treatment of drug-susceptible TB (DS-TB) and rifampicin-resistant/multidrug-resistant-TB (RR/MDR-TB); infection control; management of the TB-exposed neonate; feeding considerations; counseling and support strategies; and treatment of TB infection.

## METHODS

We reviewed the literature on TB and pregnancy with a comprehensive search of the following databases: PubMed, OVID, Medline, Clinicaltrials.gov, the Cochrane Database of Systematic Reviews, and Drug/Lactation databases between January 1, 1996 and September 30, 2022. We also searched conference proceedings from TB-focused, HIV, and pulmonary conferences, international TB guidelines issued by the WHO and national TB guidelines from South Africa, the United States, Canada, the European Union and India over the same time period. We used the search terms ‘tuberculosis,’ ‘pregnancy,’ ‘fetus,’ ‘breastmilk’ and ‘treatment’. Because there are limited published data on TB and pregnancy, we also searched for unpublished policy documents on pregnancy and TB, as well as conference presentations and abstracts. If there were no published documents to review, we generated best practice statements by consulting a team of health professionals and researchers focused on TB and pregnancy identified through the Sentinel Project on Pediatric Drug-Resistant Tuberculosis (https://sentinel-project.org/). The process used for generating best practices differed from that used by some normative bodies (e.g., WHO) as it considered clinical experiences, and its target audience is clinical providers. The authors of this review have decades of experience in caring for pregnant people with TB in a myriad of settings on six continents. Some important topics in the management of pregnancy and TB were beyond the scope of this review, including management of comorbidities (i.e., HIV, diabetes mellitus)^14^–^16^ and the care of infants/children with TB disease.

## BEST CLINICAL PRACTICE FOR PREGNANT PEOPLE WITH TB

### Screening and diagnosis

Because pregnancy puts people at risk for TB and timely diagnosis is key to reducing parental and fetal risks,^17^ it is a best practice to routinely screen all pregnant people for TB (especially in high TB burden countries) at each encounter with the healthcare system through integrated systems of care.^18^ Screening can include symptom review, as well as routine submission of sputum for testing using a WHO-recommended rapid molecular diagnostic test such as Xpert^®^ MTB/RIF (Cepheid, Sunnyvale, CA, USA),^19^,^20^ as well as mycobacterial culture. TB symptom screening may not be as sensitive during pregnancy,^21^ as TB symptoms overlap with symptoms and signs of normal pregnancy.^22^ Furthermore, the accuracy of TB screening and testing can be affected by HIV status,^23^ another compelling reason for routine HIV testing among pregnant persons.^24^ For pregnant people living with HIV, urine liporarabinomannan (LAM) screening for TB can also be done following WHO recommendations. Interferon-gamma releasing assays (IGRAs) and the tuberculin skin test (TST) may be helpful in confirming exposure to TB.^25^ However, these tests may have reduced sensitivity in pregnant persons, and thus should not be relied upon as the sole basis for TB diagnosis/exclusion. Enhanced screening should continue into the immediate postpartum period because the increased TB risk persists.^26^ In terms of TB diagnosis, the same modalities and methods used to diagnose TB can be used during pregnancy. Chest radiography should be done as needed,^27^ with lead shielding of the abdomen/reproductive organs, if available. Pregnancy is not a reason to deny access to necessary diagnostic procedures, as plain chest radiographs do not expose the developing fetus to significant amounts of radiation (even in the first trimester of pregnancy).

### Reproductive health services and family planning

People who can become pregnant and who are on treatment for TB should be tested to see if they are pregnant at the time of diagnosis; screened for pregnancy at each TB-related visit; and offered pregnancy testing and referral for reproductive health services/family planning as part of their TB care.^28^,^29^ If a person being treated for TB becomes pregnant, they should be offered counseling regarding the possible risks and outcomes and services that fit with their needs and desires regarding the pregnancy (i.e., continuation or termination). This should be provided free of charge and free of judgment as part of routine TB services. Care should be taken to avoid contraceptive methods that could interact with anti-TB agents (i.e., the effectiveness of oral hormonal contraception may be decreased by several medications, e.g., the rifamycins).^30^ Close coordination between maternal-child health services and TB services can facilitate referral and care of all people of reproductive age being treated for TB.

### Treatment of DS-TB

Principles of DS-TB treatment in pregnant people should follow those in non-pregnant people. Pregnancy, however, significantly alters human physiology and metabolism of drugs.^31^ This may result in changes in drug absorption, distribution and clearance of TB drugs.^32^,^33^ Table 1 lists the commonly used first-line medications and best practices for their use in pregnancy.^34^,^35^ Most studies on the treatment of DS- TB in pregnancy show successful TB treatment outcomes and maternal, pregnancy and infant outcomes. The safety of the fluoroquinolones (FQs) during pregnancy is reviewed below.

**Table 1:** First-line TB medications and best practices for use in pregnancy.

Medication	Best practice for use in pregnancy^64^
Isoniazid	Best practice: INH is safe to use during pregnancy, but the patient should be clinically monitored for signs and symptoms of liver toxicity. For patients using INH, monitoring of liver function can be considered monthly, especially for those with a history of liver disease. Liver function should be assessed in anyone with symptoms.^65^ INH should be administered with vitamin B6 supplements and neuropsychiatric symptom screening.
Rifampin	Best practice: RIF is safe to use in pregnancy, but clinicians may want to encourage supplementary vitamin K to prevent anemia in newborns and the parent.
Rifapentine	Best practice: RPT use is likely to be safe in pregnancy, but higher rates of clearance have been seen in people living with HIV on efavirenz-based ART. The clinical significance of this is unclear, but currently does not require a dose adjustment.
Pyrazinamide	Best practice: It is not known whether PZA crosses the placenta; widespread global use has shown that PZA is safe to use during pregnancy. The patient should be clinically monitored for signs and symptoms of liver toxicity. For patients using PZA, monitoring of liver function can be considered monthly, especially for those with a history of liver disease. Liver function should be assessed in anyone with symptoms.
Ethambutol	Best practice: EMB is safe to use in pregnancy
Moxifloxacin	Best practice: Based on limited information during pregnancy, MFX appears safe to use in pregnancy,^66^ but monthly electrocardiogram monitoring may be considered to assess for QTc prolongation in those with cardiovascular risk factors (i.e., family history of sudden death, baseline arrhythmia).^67^

INH = isoniazid; RIF = rifampicin; RPT = rifapentine; ART = antiretroviral therapy; PZA = pyrazinamide; EMB = ethambutol; MFX = moxifloxacin.

### Treatment of RR/MDR-TB

The treatment of RR/MDR-TB during pregnancy is generally associated with good outcomes for the pregnant person and the neonate, although data are limited.^36^,^37^ Treatment should be initiated as rapidly as possible once an RR/MDR-TB diagnosis has been made and the most effective regimen possible should be used.^38^ There are limited data regarding the use of many of the second-line drugs in pregnant people,^39^ as there has not been fair inclusion in most RR/MDR-TB clinical trials, including those establishing the safety and efficacy of all-oral regimens.^40^ This is unfortunate as the older standard regimens (e.g., containing ethionamide or injectable agents) are toxic to both pregnant people and their fetus.^41^ However, there are emerging data from cohort studies that demonstrate the safety of many second-line drugs in pregnancy.^42^–^44^ Table 2 summarizes the commonly used second-line medications for treating RR/MDR-TB in pregnancy and also includes information on their safety profiles and suggested monitoring strategies for use. Regimen construction in pregnant people follows a similar approach as in non-pregnant people: selecting 4–5 drugs that are active against the person’s particular TB strain is desirable. Clinicians should prioritize the use of the WHO Group A medications (bedaquiline [BDQ], linezolid [LZD], and the third-generation FQs)^45^,^46^ and the WHO Group B medications (clofazimine [CFZ] and cycloserine [CS}).^47^,^48^ The novel nitroimidazole agent, pretomanid, has been associated with testicular toxicity in animal studies and the US Food and Drug Agency has mandated semen studies in humans prior to recommending the drug for broader use. Injectable agents such as amikacin should be avoided unless needed for rescue regimens, as they can cause permanent hearing loss in both pregnant people and developing babies due cranial nerve VIII damage related to cumulative dose. Both ethionamide and prothionamide can induce nausea and vomiting, which can worsen the hyperemesis associated with pregnancy. Additionally, these drugs have the potential to be hepatotoxic and can cause neural tube defects, although the mechanism for the latter is not entirely clear and is not dose-dependent. Therefore, it is advisable to avoid using these medications during pregnancy unless they are required for rescue regimens.

**Table 2:** Second-line TB medications and best practices for use in pregnancy.

Medication	Pregnancy best practice	Monitoring and support
WHO Group A medications		
Bedaquiline	Proven safe in small cohorts. Can be used but may be associated with lower birth weight of babies	ECG monitoring for QTc prolongation at baseline and every 4 weeks thereafter. More frequent monitoring may be needed in persons with a history of cardiac arrhythmias or with symptoms of syncope or ischemia. Fetal monitoring for growth abnormalities.
Levofloxacin/Moxifloxacin	Proven safe in small cohorts and can be used but may be associated with lower birth weight of babies	ECG monitoring for QTc prolongation at baseline and every 4 weeks thereafter if receiving MFX. More frequent monitoring may be needed in persons with a history of cardiac arrhythmias or with symptoms of syncope or ischemia. Fetal monitoring for growth abnormalities.
Linezolid	Proven safe in small cohorts and can be used but associated with bone marrow suppression and anemia. Monitor hemoglobin and complete blood count regularly (i.e., at baseline, Week 2, and then monthly while on LZD).	Monthly complete blood count (more frequent hemoglobin checks if anemia is detected). Monthly visual acuity testing. Monthly peripheral neuropathy screening.
WHO Group B medications		
Clofazimine	Proven safe in small cohorts and can be used. However, the parent must be counseled about skin discoloration in themselves and their newborn.	May lead to reversible hyperpigmentation in pregnant persons and neonates, which may take weeks to resolve. ECG monitoring for QTc prolongation at baseline and every 4 weeks after More frequent monitoring may be needed in persons with a history of cardiac arrhythmias or with symptoms of syncope or ischemia.
Cycloserine/terizidone	Proven safe in small cohorts and can be used	Give with vitamin B6 supplementation. Screen for neuropsychiatric symptoms.
WHO Group C medications		
Delamanid	Proven safe in very small cohorts and can be used	ECG monitoring for QTc prolongation at baseline and every 4 weeks thereafter. More frequent monitoring may be needed in persons with a history of cardiac arrhythmias or with symptoms of syncope or ischemia.
Amikacin	Aminoglycoside use is associated with damage to fetal inner ear and to the hearing of the pregnant person and should be avoided during pregnancy. AMK use is also associated with damage to the fetal kidneys along with eight cranial nerve damage associated with aminoglycoside exposure.	Can be considered if there is no other option and the life of the pregnant person is at risk. Must obtain baseline audiometry and monthly audiometry for pregnant person. Of note, toxicity risk also applies to the developing fetus in terms of renal damage and cranial nerve VIII damage.
Ethionamide	Associated with neural tube defects and must be administered with prenatal multivitamins. Can exacerbate nausea and vomiting during pregnancy May increase the risk of hepatotoxicity, especially in the third trimester. Can be associated with hypothyroidism. If used TSH should be monitored monthly.	Can be considered if there are limited treatment options. Monitor TSH at baseline and every 1–3 months. Give with folate, thiamine and vitamin B6 supplementation (i.e., a prenatal vitamin). May be associated with nausea and vomiting which could worsen the emesis often seen in early pregnancy.
Para-aminosalicylic acid	Can exacerbate nausea and vomiting during pregnancy. Can be associated with hypothyroidism and TSH should be monitored monthly if used.	Monitor TSH at baseline and every 1–3 months. Can be considered if there are limited treatment options. May be associated with nausea and vomiting, which could worsen the emesis often seen in early pregnancy.
Imipenem (or meropenem) in combination with amoxicillin-clavulanic acid	Proven safe in small cohorts and can be used.	Requires placement of IV line for administration over prolonged periods of time, although they can be administered through a normal IV cannula for shorter periods of time. AMX/CLV must be given orally 30 min prior or the carbapenem will not be active against *M. tuberculosis*.
Other		
Pretomanid*	The manufacturer and most national and international guidelines make no recommendations on the use of PMD in pregnancy and the drug has not been given during pregnancy. Animal studies showed testicular toxicity in mice and human semen studies are currently being analyzed.	Delamanid should be used as the nitroimidazole of choice if a medication from this class of agents is needed.

* Pretomanid was not included in the WHO guidelines drug grouping and has only been assessed as part of standard combination regimens. ECG = electrocardiogram; LZD = linezolid; AMK = amikacin; TSH = thyroid stimulating hormone; IV = intravenous; AMX/CLV =amoxicillin-clavulanic acid; PMD = pretomanid.

In terms of therapy duration, although pregnant people have not been included in most studies of all-oral shorter regimens, there is no reason why they should not benefit from shorter regimens containing drugs that are not contraindicated during pregnancy. One notable study of a shorter, all-oral regimen that included seven pregnant people is the Building Evidence for Advancing New Treatment (BEAT) Tuberculosis study from South Africa (NCT 04062201),^49^ assessing the efficacy of an all-oral regimen containing BDQ, delamanid [DEL], LZD, and either levofloxacin [LFX] or clofazimine [CFZ] administered for 6 months. The experimental regimen was non-inferior to the standard of care, and this is thus an option for an all-oral shorter regimen for pregnant people with RR/MDR-TB. People who are pregnant are at high-risk for treatment interruptions during labor and delivery. This is often due to fear-based infection control practices, as well as lack of access to TB medications (especially second-line drugs) within obstetric facilities. For this reason, it is best clinical practice to issue pregnant people an “emergency” supply of their TB medications to take during and after delivery and to provide referral letters documenting their medication needs before their estimated due date. Nutritional supplementation should also be strongly considered for pregnant people on treatment for RR/MDR-TB. At a minimum, this should consist of a prenatal vitamin and caloric supplementation with food. Programs should decide on the optimal nutritional support packages based on local foods and practices.

### Compassionate infection control practices

Most pregnant people who are on effective therapy for TB are unlikely to transmit TB,^50^ provided they remain on and complete such therapy during their pregnancy, delivery and post-partum period. They should thus be attended to with routine frequency during labor and delivery, following standard infection control practices for individuals giving birth. Furthermore, infection control measures should not typically involve the separation of a parent from their newborn. Such separation is rarely justified and can have negative consequences for both the parent and the child. The risk of transmission to others, including newborns, is highest if the pregnant person is not on effective therapy, not engaged in care, or struggling with medication adherence. Fear-based infection control practices may increase feelings of stigma and shame and could inadvertently lead to struggles with adherence that might result in increased risk of transmission. Thus, it is recommended that pregnant people with TB be treated with dignity and respect in a supportive environment as the most effective infection control practice. There may be some instances during which differentiated infection control strategies are needed, as summarized in Table 3.

**Table 3:** Infectious control methods for use with parent and neonate.

TB status of the pregnant or postpartum person	Health status of the infant/newborn	Infection control best practices
If any of the following is present: Treatment for RR/MDR-TB was started less than 2 weeks ago; Treatment is possibly ineffective (i.e., there is known or likely resistance to the WHO Group A or B medications); The person being treated remains smear-positive after baseline; The person being treated has current extensive, cavitary disease; The person being treated is clinically deteriorating; The person being treated has experienced challenges with adherence and may be incompletely treated	If any of the following is present The infant is immunocompromised; The infant is premature (born before 37 weeks gestational age); The infant is very underweight (weighs less than 1,500 g at birth)	Patient should be referred for necessary medical, psychological and social support services to optimize treatment regimens and access to necessary medical care, and to reinforce and facilitate good adherence to treatment since treatment adherence and completion is the best way to ensure the health of the pregnant person and the child Patient and family should be educated/counseled/reminded of the importance of adequate ventilation – i.e., opening windows and doors, spending time outside where possible, etc. Pregnant/postpartum person should wear an N95 mask (if available; if not, a surgical mask) whenever possible when around others, including when in close contact with infant (i.e., during holding or feeding)
If all of the following are present: Treatment for RR/MDR-TB was started ≥2 weeks ago; Treatment is likely to be effective; The most recent smear of the person being treated is negative, if available; The person being treated is likely to be adherent	If all of the following are present: The infant is gaining weight; The infant is on effective treatment for their immunocompromising condition (if such conditions are present); The infant is on effective RR-TB preventive therapy, if indicated	Medical, psychological and social support services to optimize treatment regimens and access to necessary medical care should continue to reinforce and facilitate good adherence to treatment Ventilation should continue to be optimized Pregnant/post-partum person does not need to wear an N95 or surgial mask, as long as they are able to adhere to an effective treatment regimen

RR/MDR-TB = rifampin/multidrug-resistant TB.

### Feeding considerations

Most TB occurs in regions of the world where infant formula feeding is not affordable, feasible, acceptable, sustainable, or safe. Breastmilk is best for neonates in most settings, and there is no absolute contraindication to breastfeeding if the peri-partum person is being treated for TB. Few studies have been done to assess if anti-TB medications are present in breastmilk and may pass to the infant, and this is a key research priority. Table 4 presents what is known about breastmilk and anti-TB medications, although much of this is inferred based on drug properties and/or modeling studies as opposed to actual assessments of drug concentrations in the breastmilk or infant. Some people may prefer not to breastfeed their infants or may be unable to do so for a variety of reasons. In such settings, the parent should be supported to offer alternative feeding to the baby and provided with formula supplies with advice on how to safely prepare the formula to administer to the infant. Whatever feeding strategy is chosen, the parent should be supported to complete therapy.

**Table 4:** TB medications and breastfeeding considerations.

Medication	Breastfeeding considerations^68^,^69^
First-line medications	
Isoniazid	Studies demonstrate it is present in the breastmilk,^70^ but likely in concentrations that do not exceed the recommended doses in infants
Rifampin	Single oral dose study from 1969,^71^ and no direct measurements from breastfed infants. Modelling suggests low concentrations in breastmilk, and infant exposure likely to be low. People on this medication should be counseled that it may lead to discoloration of the breastmilk
Rifapentine	Low concentrations in breastmilk,^72^ but likely do not exceed the recommended doses in infants. No direct measurements from breastfed infants. People on this medication should be counseled that it may lead to discoloration of the breastmilk
Pyrazinamide	Low concentrations have been measured in breastmilk,^73^ but likely do not exceed the recommended doses in infants. No direct measurements from breastfed infants
Ethambutol	Low concentrations have been measured in breastmilk,^73^ but likely do not exceed the recommended doses in infants. No direct measurements from breastfed infants, but physiologically based pharmacokinetic modelling suggests infant exposure unlikely to be significant^74^
Moxifloxacin	No human data exist on this drug and breastmilk concentrations. It is likely present in the breastmilk, but likely in concentrations that likely do not exceed the recommended doses in infants
Second-line medications	
Bedaquiline	Very high concentrations in breastmilk, and the single breastfed infant who has been studied was found to have therapeutic concentrations.^45^ Theoretically this could offer protection to the infant from RR/MDR-TB infection but also places the infant at risk for toxicity. Infants should be assessed regularly for signs of bedaquiline toxicity, which include liver toxicity and QTc prolongation. This could be done through physical examination as well as checking an electrocardiogram and liver enzymes at Week 4, Week 8 and Week 12 of breastfeeding
Levofloxacin/moxifloxacin	Limited data indicate low concentrations in breastmilk for levofloxacin^75^ (and no data exist on moxifloxacin), but likely in concentrations that do not exceed the recommended doses in infants. No direct measurements from breastfed infants
Linezolid	Studies from single participants demonstrate it is present in the breastmilk^76^, but likely in concentrations that do not exceed the recommended doses in infants. A single sample from a single breastfed infant indicated an undetectable concentration,^77^ but this has not been studied systematically
Clofazimine	A study among breastfeeding women receiving clofazimine in leprosy indicated low concentrations in breastmilk.^78^ No direct measurements from breastfed infants. People on this medication should be counseled that it may lead to discoloration of the breastmilk (ranging from pink to red). This could lead to temporary skin hyperpigmentation in the infant and families should be counseled about this
Cycloserine/terizidone	Has been measured in breastmilk, but not studied systematically. Concentrations unlikely to exceed the recommended doses in infants. No direct measurements from breastfed infants
Delamanid	No human data exist on this drug and breastmilk concentrations. It is likely present in the breastmilk, but in concentrations that do not exceed the recommended doses in infants
Amikacin	Associated with hearing loss in parent and should be avoided unless necessary to save the parent’s life. Single-dose studies have indicated low concentrations in breastmilk,^79^ but this has never been studied systematically. No direct measurements from breastfed infants
Ethionamide	Has not been studied in breastfeeding parents or their infants. This agent is not advised for routine use in the treatment of RR/MDR-TB and if possible an alternative agent should be considered for breastfeeding parent. If this is not possible, breastfeeding can be supported, but the infant should be monitored for symptoms of toxicity especially if newborn or pre-term, including for thyroid toxicity and liver toxicity.
Para-aminosalicylic acid	A single-dose study in a single participant indicated a low concentration in breastmilk.^80^ This is anticipated to not exceed the recommended doses in infants. No direct measurements from breastfed infants
Imipenem (or meropenem) and amoxicillin/clavulanic acid	Low concentrations in breastmilk.^81^ May be used during breastfeeding but the infant should be monitored for symptoms of toxicity such as liver toxicity, rash and seizures. No direct measurements from breastfed infants
Pretomanid	No measurements in breastmilk or breastfed infants have been reported. Should not be used in breastfeeding parent as it may be associated with reproductive toxicity. If a nitroimidazole is needed, delamanid is likely to be a better option.

### Counselling and support strategies

Pregnancy is often an exciting but stressful time. When this period is complicated by TB, it can lead to added emotional distress for pregnant people and their families.^51^ TB also can lead to catastrophic costs for people living with the disease,^52^ in addition to the socio-economic implications of pregnancy. Thus, it is imperative that pregnant people and their families be given socio-economic and psychological support throughout the course of treatment. The precise packages of socio-economic support (e.g., transportation vouchers, food, conditional cash transfers) should be determined by the needs of the pregnant person, program resources, and support available from other organizations (i.e., non-governmental organizations, faith-based groups). Many pregnant people report feeling isolated and stigmatized when they find out they have TB, something that is often inadvertently perpetuated by healthcare providers.^8^ Even sympathetic providers may exacerbate feelings of anxiety and depression if they convey that ‘little is known’ about the risks of TB treatment during pregnancy. Although it is important to be honest when communicating the lack of high-quality data from large populations of pregnant people undergoing TB treatment, providers can be reassuring and supportive. It is important to continue screening for mental health symptoms throughout the pregnant and postpartum period and refer to psychologic or psychiatric services when needed. Both TB infection and pregnancy are risk factors for depression. Postpartum depression is common and underdiagnosed,^53^ and can lead to treatment non-adherence and suicidal ideation.

Treatmentofall forms ofTB during pregnancy should involve nutritional supplementation as well, especially when treatment requires FQs or BDQ as these two drugs may be associated with lower birth-weight infants. Multivitamins designed for pregnancy and nutritious food supplementation should be routinely provided, as should transportation support.

### Pregnancy and treatment of TB infection

Because pregnant people are at higher risk of having poor pregnancy and TB-related outcomes if they develop TB disease, the treatment of TB infection – commonly referred to as TB preventive therapy (TPT) – is an important health priority. There are some key considerations in the routine offer of TPT during pregnancy. The WHO recommends that all pregnant people should undergo HIV testing as part of routine antenatal care, and those who are living with HIV should be offered immediate TPT (once active TB disease is ruled out) as it has a clear benefit in this population.^54^ However, among pregnant people who are not living with HIV, the decision to offer routine, immediate TPT is more controversial. This is because data from two trials suggested an increased risk of adverse pregnancy outcomes using isoniazid (INH, H) preventive therapy after the first 14 weeks of pregnancy. The first of these was the “TB APPRISE” study (International Maternal, Pediatric, Adolescent AIDS Clinical Trials Network [IMPAACT] study P1078; NCT01494038), which was a randomized controlled trial assessing an immediate vs. delayed strategy for a 6-month INH TPT regimen among pregnant people living with HIV. The trial enrolled 956 pregnant people and found similar rates of TB in both groups, but a higher rate of composite adverse pregnancy outcomes (including stillbirth, low birth weight, pre-term delivery, and congenital abnormalities) among those initiating TPT immediately vs. those whose TPT was delayed until 12 weeks after delivery.^55^ A preliminary analysis of the pharmacokinetic data from this study suggests that adverse pregnancy outcomes may be driven by INH metabolites.^56^ To further clarify the risks seen in TB APPRISE, an analysis of the pregnancies that occurred in the INH arm of another TPT trial (Brief Rifapentine-Isoniazid Evaluation for TB Prevention, ACTG 5279, NCT 01404312) was undertaken, which found that when INH TPT was given in the first trimester pregnancy, an increase in non-live births was seen.^57^ However; a systematic review and meta-analysis of studies using INH TPT in pregnancy showed inconsistent associations with INH TPT and adverse pregnancy outcomes, leading the authors to conclude that “given the grave consequences of active TB during pregnancy” TPT should not be deferred.^58^ A smaller study (50 participants) of a 3-month TPT regimen consisting of once weekly INH and rifapentine (3HP) aimed at assessing dosing and safety during pregnancy did not report any maternal or infant serious adverse events (IMPAACT Network Study 2001, NCT 02651259).^59^ A larger study of 1 month of daily HP vs. 3 months of weekly HP during pregnancy will begin enrollment in 2023 (NCT05122026) and could provide the evidence for preferentially offering either 1HP or 3HP over longer INH TPT regimens. In all instances, the final decision about whether or not TPT should be taken during pregnancy should be based on a discussion of the possible benefits and risks with the pregnant person (and anyone they choose to involve from their support networks) while taking into account their values and hopes for the pregnancy as well as the gestational age of the pregnancy, the degree of immunosuppression, and other individual risk factors.

### Research considerations

There are many areas in which more research is required to better address the many needs of pregnant people living with TB. There are three main ways in which additional evidence could be collected on the care of pregnant people with TB.^60^ First, through studies specifically conducted in pregnant and post-partum people. This could include studies of drug dosing and efficacy, as well as regimen tolerability and feasibility. Studies should also be done to document the needs and experiences of pregnant people along the treatment spectrum. Second, it is important to consider the inclusion of pregnant individuals in ongoing TB studies, particularly in cases where there is no evidence indicating that the drugs being studied pose a risk to either the pregnant person or the fetus. Finally, people who are already enrolled in TB studies who become pregnant should be allowed to continue on the study if they provide consent. These methods of data collection could be combined with enhanced pregnancy registers to provide more robust evidence for the optimal treatment of pregnant people with TB.

## CONCLUSION

Pregnant people living with TB face multiple unique challenges and merit special attention to help manage their TB. The journey of a pregnant person with TB and the barriers they might face is shown in the Figure, which also highlights key “best practices” that could improve the care of a pregnant person throughout the treatment process. At all timepoints, it is essential that both TB and reproductive care be provided in a way that people welcome, feel cared for and confident to complete their treatment and to manage their pregnancy in ways that best serve their needs. By enhancing the methods to diagnose and treat TB during pregnancy with an understanding of the experiences and needs of pregnant TB patients, we can provide optimal care of parents and their neonates. There is an urgent need for fair inclusion of pregnant people early in clinical trials and studies and to expand the use of systematic pregnancy registers in the care of people with TB.^61^,^62^ Although trial data are being generated on how best to serve pregnant people, their infants and their families, much can be done now with what is currently known to improve their care. Having TB can be a difficult experience, but there is no reason for pregnant people to undergo additional stress. Compassionate and high-quality care must become the norm for all people with TB,^63^ and the best clinical practices identified here should help to provide such services during pregnancy.

**Figure:**
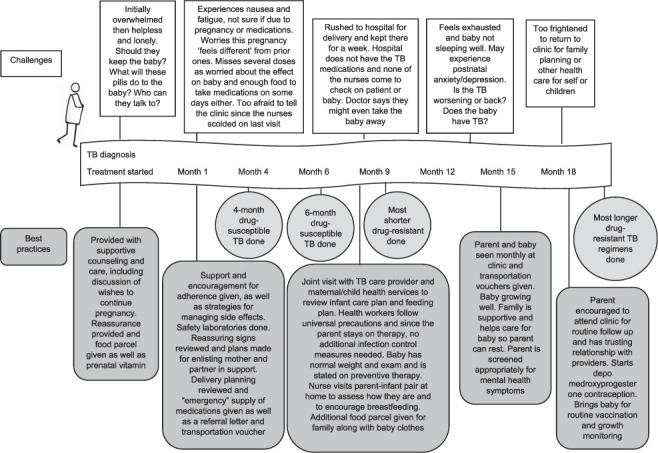
Treatment journey, barriers and best practices for pregnant people with TB

## References

[1] World Health Organization (2022). Global TB Report, 2022.

[2] Sugarman J (2014). Tuberculosis in pregnancy: an estimate of the global burden of disease. Lancet Glob Health.

[3] Grange J (2010). Tuberculosis in association with HIV/AIDS emerges as a major nonobstetric cause of maternal mortality in Sub-Saharan Africa. Int J Gynaecol Obstet.

[4] Zumla A, Bates M, Mwaba P (2014). The neglected global burden of tuberculosis in pregnancy. Lancet Glob Health.

[5] Gupta A (2016). Toward earlier inclusion of pregnant and postpartum women in tuberculosis drug trials: consensus statements from an International Expert Panel. Clin Infect Dis.

[6] Gupta A (2019). Inclusion of key populations in clinical trials of new antituberculosis treatments: current barriers and recommendations for pregnant and lactating women, children, and HIV-infected persons. PLoS Med.

[7] Loveday M, Hlangu S, Furin J (2019). Healthcare provider discrimination toward pregnant women with rifampin-resistant tuberculosis. Emerg Infect Dis.

[8] Loveday M, Hlangu S, Furin J (2020). “Take the treatment and be brave”: care experiences of pregnant women with rifampicin-resistant tuberculosis. PLoS One.

[9] Bates M (2015). Perspectives on tuberculosis in pregnancy. Int J Infect Dis.

[10] Sobhy S (2017). Maternal and perinatal mortality and morbidity associated with tuberculosis during pregnancy and the postpartum period: a systematic review and meta-analysis. Int J Gynaecol Obstet.

[11] Dennis EM (2018). Tuberculosis during pregnancy in the United States: racial/ethnic disparities in pregnancy complications and in-hospital death. PLoS One.

[12] Mathad JS, Gupta A (2012). Tuberculosis in pregnant and postpartum women: epidemiology, management, and research gaps. Clin Infect Dis.

[13] Palacios E (2009). Drug-resistant tuberculosis and pregnancy: treatment outcome of 38 cases in Lima, Peru. Clin Infect Dis.

[14] Yaghoubi A (2020). Tuberculosis, human immunodeficiency viruses and TB/HIV co-infection in pregnant women: a meta-analysis. Clin Epidemiol Glob Health.

[15] Bekker A (2016). Tuberculosis disease during pregnancy and treatment outcomes in HIV-infected and uninfected women at a referral hospital in Cape Town. PloS One.

[16] Gupta A (2011). Maternal tuberculosis: a risk factor for mother-to-child transmission of human immunodeficiency virus. J Infect Dis.

[17] Pasipamire M (2020). Detecting tuberculosis in pregnant and postpartum women in Eswatini. Afr J Lab Med.

[18] Nguyen HT (2014). Tuberculosis care for pregnant women: a systematic review. BMC Infect Dis.

[19] Bates M (2013). Use of Xpert^®^ MTB/RIF assay for diagnosing pulmonary tuberculosis comorbidity and multidrug-resistant tuberculosis in obstetrics and gynecology inpatient wards at a university teaching hospital Lusaka, Zambia. Trop Med Int Health.

[20] Martinson N Universal sputum testing vs symptom-based testing for tuberculosis (TB) in HIV infected pregnant women: a cluster-randomised implementation trial in South Africa.

[21] Hoffmann CJ (2013). High prevalence of pulmonary tuberculosis but low sensitivity of symptom screening among HIV-infected pregnant women in South Africa. PloS One.

[22] Miele K, Bamrah Morris S, Tepper NK (2020). Tuberculosis in pregnancy. Obstet Gynecol.

[23] Gounder CR (2011). Active Tuberculosis case-finding among pregnant women presenting to antenatal clinics in Soweto, South Africa. J Acquir Immune Defic Syndr.

[24] LaCourse SM (2016). Tuberculosis case finding in HIV-infected pregnant women in kenya reveals poor performance of symptom screening and rapid diagnostic tests. J Acquir Immune Defic Syndr.

[25] World Health Organization (2022). Use of alternative interferon-gamma release assays for the diagnosis of TB infection.

[26] Odayar J (2018). Burden of tuberculosis in HIV-positive pregnant women in Cape Town, South Africa. Int J Tuberc Lung Dis.

[27] Pop LG (2021). Tuberculosis in pregnancy. J Med Life.

[28] Kosgei R (2013). Screening for tuberculosis in pregnancy: do we need more than a symptom screen? Experience from western Kenya. Public Health Action.

[29] Cornish E (2020). Improving access to contraception through integration of family planning services into a multidrug-resistant tuberculosis programme. BMJ Sexual Reprod Health.

[30] Mngqibisa R PK of dose adjusted emergency contraception with rifampicin therapy in ACTG A5375.

[31] Widen EM, Gallagher D (2014). Body composition changes in pregnancy: measurement, predictors and outcomes. Eur J Clin Nutr.

[32] Schalkwijk S, Greupink R, Burger D (2017). Free dug concentrations in pregnancy: bound to measure unbound. Br J Clin Pharmacol.

[33] Shiu J, Min A, Kiang T (2021). Clinical pharmacokinetics and pharmacodynamics of anti-tubercular drugs in pregnancy. Eur J Drug Metab Pharmacokinet.

[34] Denti P (2015). Population pharmacokinetics of rifampin in pregnant women with tuberculosis and HIV coinfection in Soweto, South Africa. Antimicrob Agents Chemother.

[35] Abdelwahab MT (2020). Population pharmacokinetics of isoniazid, pyrazinamide, and ethambutol in pregnant South African women with tuberculosis and HIV. Antimicrob Agents Chemother.

[36] Alene KA (2022). Treatment outcomes among pregnant patients with multidrug-resistant tuberculosis: a systematic review and meta-analysis. JAMA Network Open.

[37] van de Water BJ (2020). Tuberculosis clinical presentation and treatment outcomes in pregnancy: a prospective cohort study. BMC Infect Dis.

[38] Walt M (2020). Retrospective record review of pregnant women treated for rifampicin-resistant tuberculosis in South Africa. PLOS One.

[39] Acquah R (2021). Outcomes of children born to pregnant women with drug-resistant tuberculosis treated with novel drugs in Khayelitsha, South Africa: a report of five patients. Pediatr Infect Dis J.

[40] Padmapriyadarsini C (2017). Multidrug-resistant tuberculosis during pregnancy. Indian J Tuberc.

[41] Tabarsi P (2011). Standardised second-line treatment of multidrug-resistant tuberculosis during pregnancy. Int J Tuberc Lung Dis.

[42] Alene K, Jegnie A, Adane A (2021). Multidrug-resistant tuberculosis during pregnancy and adverse birth outcomes:a systematic review and meta-analysis. Br J Obstet Gynecol.

[43] Baluku J, Bongomin F (2021). Treatment outcomes of pregnant women with drug-resistant tuberculosis in Uganda: A retrospective review of 18 cases. Int J Infect Dis.

[44] Mokhele I (2021). Treatment and pregnancy outcomes of women exposed to second-line anti-tuberculous drugs in South Africa. BMC Pregnancy Childbirth.

[45] Court R (2022). Bedaquiline exposure in pregnancy and breastfeeding in women with rifampicin-resistant tuberculosis. Br J Clin Pharmacol.

[46] Jaspard M (2017). Bedaquiline and linezolid for extensively drug-resistant tuberculosis in pregnant women. Emerg Infect Dis.

[47] Loveday M (2021). Maternal and infant outcomes among pregnant women treated for multidrug/rifampicin-resistant tuberculosis in South Africa. Clin Infect Dis.

[48] Ahmed S, Lachenal N, Moodliar R, EndTB and TB-PRACTECAL Study Teams Pregnancy outcomes for patients treated with new and repurposed drugs for drug-resistant tuberculosis.

[49] Conradie F High rate of successful outcomes treating RR-TB with a delamanid-bedaquiline regimen in BEAT Tuberculosis: an interim analysis.

[50] Dharmadhikari AS (2014). Rapid impact of effective treatment on transmission of multidrug-resistant tuberculosis. Int J Tuberc Lung Dis.

[51] Kodadhala V, Gudeta A, Zerihun A (2016). Postpartum tuberculosis: a diagnostic and therapeutic challenge. Case Rep Pulmonol.

[52] Wingfield T (2014). Defining catastrophic costs and comparing their importance for adverse tuberculosis outcome with multi-drug resistance: a prospective cohort study, Peru. PLoS Med.

[53] Duko B, Bedaso A, Ayano G (2020). The prevalence of depression among patients with tuberculosis: a systematic review and meta-analysis. Ann Gen Psychiatry.

[54] World Health Organization (2020). WHO consolidated guidelines on tuberculosis. Module 1: prevention: tuberculosis preventive therapy.

[55] Gupta A (2019). Isoniazid preventive therapy in HIV-infected pregnant and post-partum women. N Engl J Med.

[56] Aaron L Pharmacokinetic-pharmacogenetic-pharmacodynamic analysis of isoniazid and efavirenz for predicting adverse pregnancy outcomes in women with HIV.

[57] Gupta A Adverse pregnancy outcomes among HIV-infected women exposed to isoniazid preventive therapy in the BRIEF-TB trial.

[58] Hamada Y (2020). The safety of isoniazid tuberculosis preventive treatment in pregnant and postpartum women: systematic review and meta-analysis. Eur Respir J.

[59] Mathad J (2022). Pharmacokinetics and safety of 3 months of weekly rifapentine and isoniazid for tuberculosis prevention in pregnant women. Clin Infect Dis.

[60] McKenna L (2017). A community perspective on the inclusion of pregnant women in tuberculosis drug trials. Clin Infect Dis.

[61] Mehta U (2018). Assessing the value of Western Cape Provincial Government health administrative data and electronic pharmacy records in ascertaining medicine use during pregnancy. S Afr Med J.

[62] Mehta U (2019). Why South Africa urgently needs to support the development of pregnancy exposure registries. S Afr Med J.

[63] Schnippel K (2016). A call to action: addressing the reproductive health needs of women with drug-resistant tuberculosis. S Afr Med J.

[64] Bothamley G (2001). Drug treatment for tuberculosis during pregnancy. Drug Safety.

[65] Kalk E (2020). Safety and effectiveness of isoniazid preventive therapy in pregnant women living with human immunodeficiency virus on antiretroviral therapy: an observational study using linked population data. Clin Infect Dis.

[66] Van Kampenhout E (2017). Pharmacokinetics of moxifloxacin and linezolid during and after pregnancy in a patient with multidrug-resistant tuberculosis. Eur Respir J.

[67] Kusmiati T (2022). Moxifloxacin concentrations correlate with QTc interval in rifampicin-resistant tuberculosis patients on shorter treatment regimens. J Clin Tuberc Other Mycobact Dis.

[68] Tran JH, Montakantikul P (1998). The safety of antituberculosis medications during breastfeeding. J Hum Lact.

[69] Loveday M, Hlangu S, Furin J (2020). Breastfeeding in women living with tuberculosis. Int J Tuberc Lung Dis.

[70] Singh N (2008). Transfer of isoniazid from circulation to breast milk in lactating women on chronic therapy for tuberculosis. Br J Clin Pharmacol.

[71] Lenzi E, Santauri S (1969). [Preliminary observations on the use of a synthetic rifamycin derivative]. Atti Accad Lancisiana Roma.

[72] Mkhize B (2022). Validation and application of a quantitative liquid chromatography tandem mass spectrometry assay for the analysis of rifapentine and 25-O-desacetyl rifapentine in human milk. J Pharm Biomed Anal.

[73] Zuma P (2022). Validation and application of a quantitative LCMS/MS assay for the analysis of first-line anti-tuberculosis drugs, rifabutin and their metabolites in human breast milk. J Chromatogr B Analyt Technol Biomed Life Sci.

[74] Partosch F (2018). Exposure of nursed infants to maternal treatment with ethambutol and rifampicin. Basic Clin Pharmacol Toxicol.

[75] Giamarellou H (1989). Pharmacokinetics of three newer quinolones in pregnant and lactating women. Am J Med.

[76] Lim FH (2017). Linezolid and lactation: measurement of drug levels in breast milk and the nursing infant. J Antimicrob Chemother.

[77] Rowe HE (2014). Transfer of linezolid into breast milk. J Hum Lact.

[78] Ozturk Z, Tatliparmak A (2017). Leprosy treatment during pregnancy and breastfeeding: a case report and brief review of literature. Dermatol Ther.

[79] Yuasa M (1974). Evaluation of amikacin in gynecological and obstetric field. Jpn J Antibiot.

[80] Holdiness MR (1984). Antituberculosis drugs and breast-feeding. Arch Intern Med.

[81] Matsuda S (1988). Pharmacokinetic and clinical studies on imipenem/cilastatin sodium in the perinatal period. A study of imipenem/cilastatin sodium in the perinatal co-research group. Jpn J Antibiot.

